# The Configurational Logic of Alliance Networks for Innovation: A Machine Learning-Enabled Investigation

**DOI:** 10.3390/e28090968

**Published:** 2026-08-30

**Authors:** Wenhao Zhou, Zhiwei Zhang, Siyu Lin

**Affiliations:** Business School, Putian University, Putian 351100, China

**Keywords:** alliance network, machine learning, configurational logic, innovation

## Abstract

Alliance network embeddedness provides firms with access to external knowledge and resources, yet its innovation implications vary across firms and network contexts. This study examines how network structure and combinations of embedding characteristics are associated with corporate innovation performance. Based on 335 firm-level observations from Chinese listed biopharmaceutical manufacturing firms, the study first identifies heterogeneous alliance network environments through community detection and K-Means clustering. Four network types are identified: dyadic, ringlike, star, and complex alliances. Classification and regression trees (CART) are then employed to extract interpretable, threshold-based decision rules linking network embedding characteristics to high and non-high innovation performance. The results show that no single network characteristic is consistently associated with innovation performance across alliance types. In dyadic alliances, moderate cooperation intensity is associated with high innovation performance, whereas ringlike alliances exhibit conditional associations involving cooperation intensity and partner centrality. Star alliances are characterized by configurations involving cooperation breadth and network position, while complex alliances exhibit more multidimensional combinations of structural and relational conditions. The findings indicate that the innovation relevance of alliance network embeddedness is network-type-specific and configuration-dependent. As a complementary robustness analysis, fuzzy-set qualitative comparative analysis broadly supports several core configurational patterns identified by CART, while also revealing alternative configurations, particularly in complex alliances. The study demonstrates that understanding alliance network embeddedness requires attention to network context, empirical thresholds, and combinations of network characteristics rather than isolated network attributes.

## 1. Introduction

Innovation increasingly depends on firms’ ability to access, combine, and recombine knowledge and resources beyond their organizational boundaries [1]. Strategic alliances provide an important mechanism for obtaining complementary technological knowledge, specialized capabilities, and market resources, making interfirm alliance networks an important context for understanding corporate innovation [2,3]. From a network perspective, however, the innovation value of an alliance is not determined only by whether a firm has external relationships, but also by how the firm is embedded in the broader network of relationships. Network embeddedness can facilitate access to diverse information and resources [4], while particular network positions may simultaneously expose firms to redundancy, coordination requirements, and constraints arising from highly embedded relationships [5]. The innovation implications of alliance network embeddedness are therefore unlikely to be uniform across firms.

This observation raises a more specific question: under what network conditions is alliance network embeddedness associated with high innovation performance? Existing research provides considerable evidence that individual network characteristics matter for innovation. For example, cooperation intensity can facilitate knowledge exchange and resource sharing, while network positions such as centrality and structural holes can provide access to valuable external resources and non-redundant information [6,7]. Research has also shown that firms can benefit from network breadth and access to heterogeneous partners. However, the empirical conclusions are not always consistent. Similar network characteristics have sometimes been associated with different innovation outcomes [8], suggesting that the innovation relevance of a particular network position may depend on the surrounding relational and structural conditions rather than on the characteristic itself.

One reason for such heterogeneous findings is that firms are not embedded in identical alliance network environments. Traditional network studies [9,10] often emphasize individual firms’ positions within an overall network or an egocentric network, but alliance networks can contain structurally distinct communities characterized by different patterns of connectivity, closure, brokerage, and centralization. The network embeddedness perspective has long emphasized the importance of relational and structural context [11,12], while research on interorganizational networks has demonstrated that different network forms generate different opportunities for knowledge access, coordination, and innovation [13,14]. This suggests that the innovation implications of a network characteristic cannot be fully understood without considering the type of network structure in which that characteristic is embedded.

A second limitation concerns the way network characteristics are commonly analyzed. Much of the existing literature examines the independent association between an individual network attribute and innovation performance [15]. Such an approach is useful for identifying the role of particular network characteristics, but it may overlook the possibility that several conditions jointly correspond to an innovation outcome. In practice, a firm’s cooperation intensity may matter differently when it is combined with a highly central partner, broad alliance coverage, or an advantageous structural position. Consequently, the relevant question is not only whether a particular network characteristic is associated with innovation, but also how multiple network embedding characteristics combine within different network structures. Recent configurational research has provided an important methodological basis for addressing such questions by examining combinations of conditions rather than isolated variables [16,17].

This configurational perspective also provides a more nuanced way to understand the apparent inconsistency in prior findings on alliance network embeddedness. For example, cooperation intensity may provide greater opportunities for knowledge exchange when interaction is sufficiently developed, but greater intensity may not necessarily correspond to higher innovation performance under every network structure [18]. Similarly, central positions and structural holes can facilitate resource access, but their value may depend on how other network conditions are configured. Prior research has discussed complementary and substitution relationships among alliance network characteristics [19,20], but these relationships may not be universal across different network contexts. Instead, they may represent context-dependent configurational associations.

Although configurational approaches such as fuzzy-set qualitative comparative analysis (fsQCA) have become established tools for examining such combinations [16], an additional analytical challenge remains when the explanatory variables are continuous network measures. fsQCA is particularly useful for identifying sufficient combinations of conditions and alternative pathways to an outcome, but it does not primarily focus on the empirical thresholds at which a continuous network characteristic changes its association with an outcome. Rather than simply determining whether cooperation intensity is present or absent in a sufficient configuration, it may be useful to identify whether a specific range of cooperation intensity corresponds to different innovation-performance classifications and how that threshold interacts with other network conditions.

Decision tree models provide a complementary approach to this problem [21]. CART recursively partitions observations according to empirically derived thresholds and produces interpretable hierarchical decision paths. Its value in the present study therefore does not lie in replacing fsQCA or in claiming that machine learning is inherently superior to configurational methods. Rather, CART can complement established configurational analysis by identifying threshold-based decision rules and hierarchical conditional pathways among continuous network embedding characteristics [22]. Such rules can provide a more operational representation of how firms with similar network characteristics may be classified differently according to combinations of network conditions. The subsequent use of fsQCA allows the main configurational patterns identified by CART to be examined from a different set-theoretic perspective.

Taken together, the literature suggests three related gaps. First, although previous research [23] has established the importance of alliance network embeddedness for innovation, less attention has been devoted to systematically distinguishing the heterogeneous network environments in which firms are embedded. Second, existing studies have provided substantial evidence on the individual roles of network characteristics, but the way these characteristics combine to correspond with innovation performance across different alliance network types remains insufficiently understood. Third, configurational approaches have provided important evidence on sufficient combinations, while relatively less attention has been paid to using interpretable decision rules to identify empirical thresholds and hierarchical conditional relationships among continuous network characteristics.

This study addresses these gaps by integrating community-based network typology identification with machine-learning-enabled configurational analysis. Specifically, community detection is first used to identify densely connected alliance communities, after which K-means clustering classifies these communities according to their structural characteristics. CART is then employed to identify network-type-specific decision rules associated with high and non-high innovation performance. Rather than interpreting these rules as causal effects, the study treats them as configurational and predictive associations. Finally, fsQCA is employed as a complementary robustness approach to examine whether the principal configurational patterns are supported when the data are analyzed through a different methodological logic.

Given the exploratory and data-driven nature of the research design, the study does not impose highly specific directional hypotheses concerning individual network characteristics. Such hypotheses would require predetermined assumptions about the direction or threshold effects of particular network variables, whereas the central purpose of the analysis is to identify these conditional thresholds and combinations empirically. Instead, the study is guided by three research questions:

RQ1: What distinct types of alliance network configurations can be identified based on firms’ network embedding characteristics?

RQ2: How do the configurational associations between network embedding characteristics and innovation performance differ across alliance network types?

RQ3: What alternative configurational pathways are associated with high and non-high innovation performance within different alliance network types?

These questions correspond to the three analytical stages of the study: identifying heterogeneous alliance network types, extracting interpretable decision rules within each type, and evaluating the robustness of the identified configurational patterns.

The study contributes to the literature in three related respects. First, it shifts attention from isolated network characteristics toward a network-type-specific configurational perspective, highlighting that the innovation relevance of network embeddedness depends on the structural context in which firms are embedded. This provides a more differentiated explanation for heterogeneous findings in the alliance-network and innovation literature. Second, it demonstrates how decision-tree analysis can provide additional information for configurational research by identifying empirical thresholds and hierarchical decision pathways among continuous network characteristics. This complements rather than replaces established methods such as fsQCA. Third, by combining network typology identification with decision rules and cross-method robustness analysis, the study provides a more detailed empirical account of why similar forms of network embeddedness may correspond to different innovation-performance outcomes across firms.

The remainder of the paper is organized as follows. Section 2 presents the theoretical framework of the study. Section 3 describes the data, variable measurement, and analytical methods. Section 4 reports the empirical results, including alliance network typology, decision-rule analysis, and robustness tests. Section 5 discusses the findings in relation to previous research. Section 6 presents the main conclusions, implications, and limitations and directions for future research.

## 2. Theoretical Framework

### 2.1. Network Embeddedness

Network embeddedness provides an important theoretical perspective for understanding how firms obtain and utilize external knowledge and resources through interorganizational relationships. Granovetter [24] emphasized that economic actions are embedded in social structures and that organizational outcomes depend on both the position of actors within networks and the nature of their relationships. In alliance networks, embeddedness enables firms to access knowledge, information, and complementary resources beyond organizational boundaries, thereby influencing their innovation activities [25,26]. Accordingly, network embeddedness reflects multiple dimensions of a firm’s position and relationships rather than a single network attribute.

Existing research commonly distinguishes between structural embeddedness and relational embeddedness [4,27]. Structural embeddedness concerns the position and pattern of connections within the broader network. Structural holes can provide access to non-redundant information and facilitate knowledge recombination [12,28], while central network positions can improve access to information and resources. Relational embeddedness emphasizes the breadth and intensity of interfirm relationships. Broad collaboration can expose firms to heterogeneous knowledge, whereas intensive relationships can facilitate trust and the exchange of complex knowledge [29,30]. In this study, structural embeddedness is represented by partner centrality, closeness centrality, and structural holes, while relational embeddedness is captured by cooperation breadth and cooperation intensity.

Importantly, the innovation implications of network embeddedness are not necessarily uniformly positive. Structurally advantageous positions may provide valuable information while also generating coordination or information-processing demands; similarly, intensive or broad relationships may facilitate knowledge access but can also increase dependence, redundancy, or resource dispersion [5,29]. Previous research has therefore identified both complementary and substitution relationships among network characteristics [19,20]. The value of a particular network characteristic may consequently depend on how it interacts with other structural and relational conditions. This suggests that understanding the innovation implications of network embeddedness requires attention not only to individual network characteristics but also to their combined configuration and the broader network context in which they are embedded.

### 2.2. Alliance Network Types

Alliance networks differ in how actors are connected and how knowledge and resources circulate among them. Network structure determines the accessibility, diversity, and redundancy of resources available to firms, thereby creating different environments for knowledge exchange and innovation [9]. From this perspective, the innovation value of a firm’s network embeddedness cannot be understood independently of the structural environment in which its relationships are situated.

Network communities provide a useful perspective for capturing these differences. A community consists of a group of densely interconnected actors within a larger network [31]. Such structures reflect not only a firm’s direct relationships but also the broader relational environment through which knowledge and resources can reach the focal firm. Communities with different patterns of connectivity, closure, centralization, and path structure may therefore generate different opportunities for resource access and knowledge recombination [32,33]. More densely connected structures may facilitate repeated interaction and efficient knowledge diffusion, whereas more open or structurally differentiated environments may provide access to heterogeneous and non-redundant information. At the same time, these structures may also differ in their exposure to redundancy, coordination requirements, and dependence on particular network actors.

Accordingly, alliance network types should not be viewed simply as descriptive categories of network topology. Rather, they represent different structural contexts of resource and knowledge flows within which firms’ network embeddedness operates. The same level of cooperation breadth, cooperation intensity, or structural advantage may therefore have different implications when firms are situated in different network environments [22]. This contextual perspective provides the basis for examining whether the configurational associations between structural and relational embeddedness and innovation performance vary across alliance network types.

### 2.3. Configurational Perspective

The preceding discussion suggests that the innovation implications of alliance network embeddedness depend on both the structural context of the network and the combination of different embedding characteristics. Firms simultaneously occupy structural positions and develop relationships of different breadth and intensity. Consequently, innovation performance may emerge from particular combinations of network conditions rather than from the independent effect of a single network characteristic [16,23]. This view is consistent with configurational research, which emphasizes that organizational outcomes can result from multiple interacting conditions and that different combinations may lead to similar outcomes [34,35].

A configurational perspective is particularly relevant to alliance networks because the value of one embedding characteristic may depend on the levels of other characteristics and on the structural environment in which firms are embedded. For example, the innovation relevance of cooperation intensity may vary with partner position, structural accessibility, or the breadth of collaborative relationships. Thus, rather than assuming a universally optimal level or directional effect for an individual network characteristic, this study focuses on identifying network-type-specific configurations associated with innovation performance.

**Figure 1:**
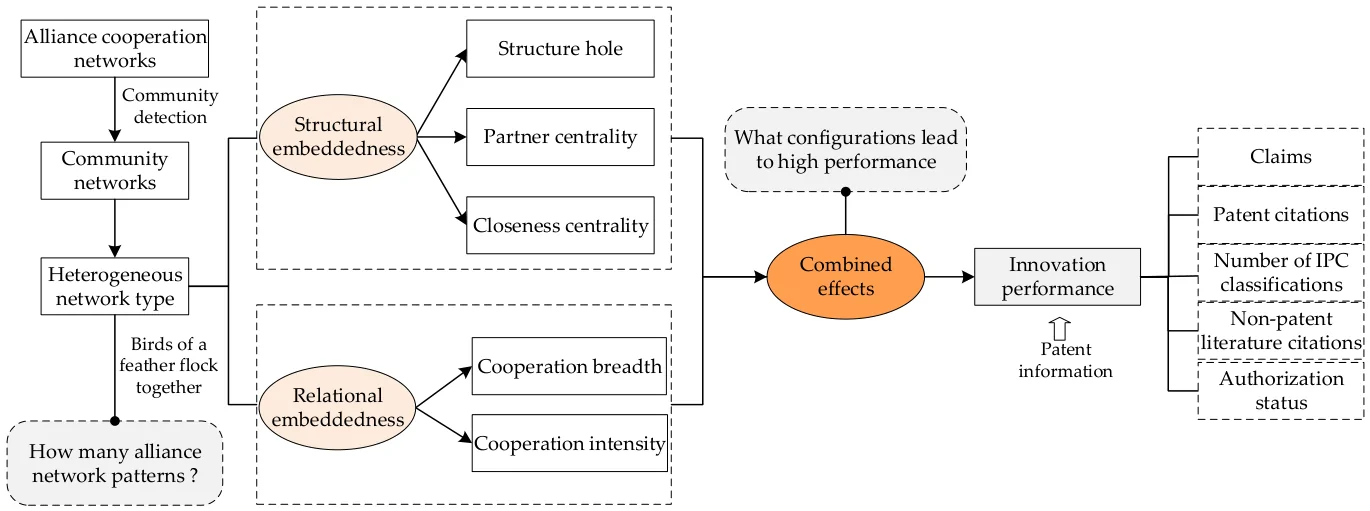
Theoretical framework of this study.

As illustrated in Figure 1, the theoretical framework integrates three parts, alliance network types, network embedding characteristics, and innovation performance. Alliance network types represent the structural context of resource and knowledge flows; network embedding characteristics capture firms’ structural and relational positions within these contexts; and innovation performance represents the resulting firm-level outcome. The framework therefore proposes that the association between network embedding and innovation performance is conditional on the broader network structure and emerges through combinations of structural and relational conditions. This configurational logic provides the theoretical basis for the subsequent empirical identification of heterogeneous alliance network types and their corresponding decision rules.

## 3. Methodology

### 3.1. Data

This study integrates data from three databases: the China Stock Market Accounting Research Database (CSMAR), which provides essential information on Chinese listed companies; the Chinese Research Data Services Platform (CNRDS), which provides corporate strategic alliance data; and the Innojoy patent database, which provides detailed patent information. The study focuses on listed companies in China’s biopharmaceutical manufacturing sector because it is a high-tech and knowledge-intensive industry with intensive R&D activities and a strong reliance on external knowledge and resources.

The data processing proceeded in four steps. First, listed firms in the biopharmaceutical manufacturing sector were identified, while firms under special treatment (ST) were excluded. Subsidiaries were consolidated with their parent companies, and firms with name changes were standardized using their securities codes to ensure consistent firm identification across databases.

Second, strategic alliance records were obtained from the CNRDS database for 2008–2020. After the above firm-level screening, 21,339 distinct cross-enterprise alliance records were retained. These records include R&D alliances, sales alliances, supply alliances, share transfers, and cooperative investments. Following the study of Schilling and Phelps [9], a three-year rolling window was used to construct ten annual alliance networks (2008–2010, 2009–2011, …, 2018–2020), and network embedding indicators were calculated using Ucinet.

Third, patent records were retrieved from the Innojoy database. Patent innovativeness was measured using citation counts, number of claims, grant status, non-patent literature references, and the first four IPC (International Patent Classification) codes. These indicators were aggregated using the entropy-weighting method. To mitigate potential reverse association, innovation performance was measured with a one-year lag. The average innovativeness of patents was used as the firm-level innovation-performance measure.

Finally, the alliance-network indicators, lagged innovation-performance measures, and other required variables were matched at the firm-year level. Observations with incomplete required information were excluded. This procedure resulted in 335 firm-level observations for the subsequent analysis.

### 3.2. Variables

#### 3.2.1. Dependent Variable

Corporate innovation performance encapsulates the efficacy of a firm’s endeavors in technological advancements, serving as an essential gauge of its output in this sphere [36]. Patents, recognized as intellectual property rights legally safeguarded, embody a repository of intricate technical knowledge and are indicative of a firm’s innovative output [37]. It is a conventional approach among scholars to assess innovative performance via the volume of invention patents. Nonetheless, the sheer number of patents inadequately reflects the innovation’s quality and its consequential influence [38]. Certain enterprises might engage in strategic behaviors, utilizing specific techniques or exploiting opportunities to amass a significant patent portfolio, which, notwithstanding, may lack genuine innovation or substantial impact on the technological sector. Consequently, this investigation employs patent quality, rather than quantity, as a metric for evaluating corporate innovation performance, thereby providing a more precise depiction of a firm’s genuine innovation contributions [39]. Patent quality is assessed through various metrics [40], and drawing upon existing literature, this study evaluates the innovativeness of a patent across five dimensions: technological coverage, technological influence, technological diversity, knowledge base, and legal protection, measured by patent claims, patent citations, IPC numbers, non-patent literature citations, and authorization status, respectively. The criteria for these indicators and their justifications are delineated below:

Claims: The count of claims indicates the breadth of legal protection afforded to the patent’s technological aspects. An elevated number of claims typically suggests a wider technological ambit covered by the patent, signifying superior patent quality [41].

Patent citations: The frequency of forward citations reflects the patent’s technological influence and its significance. A patent extensively cited by subsequent patents is deemed to exert a constructive effect on its respective domain [42].

Number of IPC classifications: The International Patent Classification system provides a uniform framework for classifying the technological fields of patents [43]. The number of four-digit IPC codes reveals the breadth of technological knowledge encompassed by the patent, indicating the diversity of technological domains it spans.

Non-patent literature citations: The volume of non-patent literature citations evidences the patent’s underlying knowledge base. A patent that incorporates references to non-patent literature is predicated on a comprehensive knowledge foundation, further attesting to its quality [44].

Authorization status: The authorization status of a patent, signifying its examination and approval, generally reflects its practical utility and legal robustness. Patents that have been authorized are likely to exhibit higher quality [45].

The significance of different patent quality metrics in assessing innovative performance may vary, with potential overlap or redundancy among some metrics [46]. Weighting these metrics can mitigate redundancy, enriching the measurement’s informational diversity. Through the analysis of patent application records from target firms and extracting data on the five patent quality metrics, this study employs the entropy weight method for weighting each quality metric, subsequently calculating the innovative value of each patent through a weighted average, as outlined in the following procedures.

Step 1: Normalization of the *j*-th indicator for the *k*-th patent.

(1) $${x^{*}}_{kj}=\frac{x_{kj}-\underset{k}{\min}\;x_{kj}}{\underset{k}{\max}\;x_{kj}-\underset{k}{\min}\;x_{kj}},\;j=1,2,\cdots ,m$$

${x^{*}}_{kj}$ represents the normalized quality indicator of the patent, which is scaled to a value between 0 and 1.

Step 2: Calculation of information entropy for the *j*-th indicator.

(2) $$E_{j}=-\frac{1}{\ln\left(n\right)}\sum_{k=1}^{n}p_{kj}\ln\left(p_{kj}\right)$$

where $p_{kj}=\frac{{x^{*}}_{kj}}{\sum_{k=1}^{n}{x^{*}}_{kj}}$, and if, then $p_{kj}\ln\left(p_{kj}\right)=0$. *n* represents the total number of patents.

Step 3: Weights assignment for the *j*-th indicator based on information entropy

(3) $$w_{j}=\frac{1-E_{j}}{m-\sum E_{j}}$$

Using the calculated weights for the patent quality indicators, these weights are applied to each patent. The patent data is matched to enterprises, which allows for the calculation of the innovative performance of focal enterprise *i* in year *t*:

(4) $$PE_{it}=\frac{1}{N_{it}}\sum_{k=1}^{N_{it}}\sum_{j=1}^{m}w_{j}\cdot patent_{i,j,k,t}$$

Here, $patent_{i,j,k,t}$ represents the *j*-th quality indicator of the *k*-th patent of focal enterprise *i* in year *t*, and $N_{it}$ is the total number of patents for *i* in year *t*.

The entropy-weighting results show that IPC numbers (0.471) and Patent Citations (0.355) receive relatively higher weights, whereas Claims (0.097), Authorization Status (0.059), and Non-patent Literature Citations (0.018) receive relatively lower weights. These differences should be interpreted as reflecting the relative information contribution and discriminatory variation of the indicators within the sample, rather than their theoretical importance to innovation performance. In particular, the relatively high weight of IPC Numbers suggests that variation in technological classification breadth provides substantial information for distinguishing patent innovativeness across observations, while the relatively high weight of Patent Citations indicates considerable cross-sectional differentiation in subsequent technological influence. The lower weights of Non-patent Literature Citations and Authorization Status indicate comparatively limited discriminatory variation in the present sample. Nevertheless, all five indicators are retained because they capture complementary dimensions of patent quality.

#### 3.2.2. Independent Variable

For the explanatory variables, this study delves into two pivotal dimensions: structural embeddedness and relational embeddedness. Structural embeddedness is articulated through the network’s structural attributes, including structural hole [47], closeness centrality [48], and partner’s centrality [49]. Relational embeddedness is encapsulated by two relational dimensions, cooperation intensity and cooperation breadth [50].

Structural hole (*SH*) represents a unique configuration characterizing non-redundant connections among actors, with firms positioned within these structural voids often perceived to have an informational control advantage. Following the established research [18], this study employs the constraint measure to quantify the structural holes for the focal firm *i* as follows:

(5) $$SH=2-{\left(P_{ij}+\sum_{q}P_{iq}P_{qj}\right)}^{2}$$

where *P_ij_* denotes the direct relational investment of focal firm *i* towards *j*, and *P_iq_* represents the relational investment of the relationship between node *i* and *q*.

Closeness centrality (*CC*) is a critical metric for a node’s central position within a network. A focal firm exhibits higher closeness centrality when the sum of the shortest paths between it and all other reachable nodes is minimized, suggesting proximity to the network’s core and superior efficiency in knowledge transmission. Its calculation is as follows:

(6) $$CC_{i}=\frac{1}{\sum_{j\notin i}dist\left(j,i\right)}$$

where $dist\left(j,i\right)$ is the shortest path between the node *i* and *j*.

Partner’s centrality (*PC*) measures the significance of the nodes connected to the focal firm. A higher centrality of the focal firm’s partners indicates easier access to second-order capital within the network. Drawing from existing studies [49], a node’s centrality is proportionally related to the centrality of its connected nodes, satisfying the following relation:

(7) $$\lambda x_{it}=\sum_{j=1}^{n}A_{ijt}x_{jt},i=1,2,\cdots ,n$$

where *n* is the number of firms in the network. $A_{ij}$ denotes the connection between two distinct nodes *i* and *j* in a specific year *t*, with $A_{ij}=1$ if there is a link and $A_{ij}=0$ otherwise. Partner’s centrality is represented by the largest non-negative eigenvalue λ.

Cooperation breadth (*CB*) refers to the count of unique partners a focal firm collaborates with, mirroring the partner diversity within the alliance network. Higher cooperation breadth indicates access to a more heterogeneous set of social capital, affording the focal firm enhanced opportunities for innovation activities.

Cooperation intensity (*CI*) signifies the average frequency of interactions between a firm and its partners within the alliance network [51]. Cooperation intensity is quantified by the ratio of the number of interactions a focal firm has with its partners to the number of partners. For instance, in an alliance network, a focal firm with three heterogeneous partners engaging in six alliances within a period would have a cooperation intensity of 6/3 = 2.0.

#### 3.2.3. Other Variables

This paper also contemplates the differentiated impact of network-level topological structures on corporate innovation, categorizing the types of alliance networks in which firms are embedded. Essentially, this involves a heterogeneity classification of the topological structures of community networks where the firms are situated. Initially, leveraging the Louvain community detection algorithm, potential heterogeneous subgroups within the alliance networks are identified, characterized by the minimal similarity amongst themselves. Subsequently, the overall network metrics for these subgroups are computed, including network size, average clustering coefficient, network density, network diameter, and average path length. The relevant metrics and the definitions of all variables involved in this study are presented in Table 1.

### 3.3. Methods

#### 3.3.1. Social Network Analysis

Within the context of open innovation, social network analysis has emerged as a potent research tool that garners extensive attention from scholars in social science fields [52]. It is utilized to comprehend various types of relational data and complex topological structures [53]. The allure of sophisticated visualizations and the intuitive calculation of metrics facilitate an enhanced understanding of the intricate relationships and cooperative patterns among enterprises, exemplified by Granovetter’s theory of weak ties [11], Burt’s theory of structural holes [12], and Watts’s small-world model [54]. Beyond basic visualization and analysis of network characteristics, an often-overlooked aspect of social network analysis, community detection has been applied to the analysis of inter-firm alliance network embeddedness in this study. The aim of community detection is to identify network nodes with similar connections and structures, revealing potential hidden cooperation patterns. Firms within the same community usually exhibit a certain degree of similarity.

**Figure 2:**
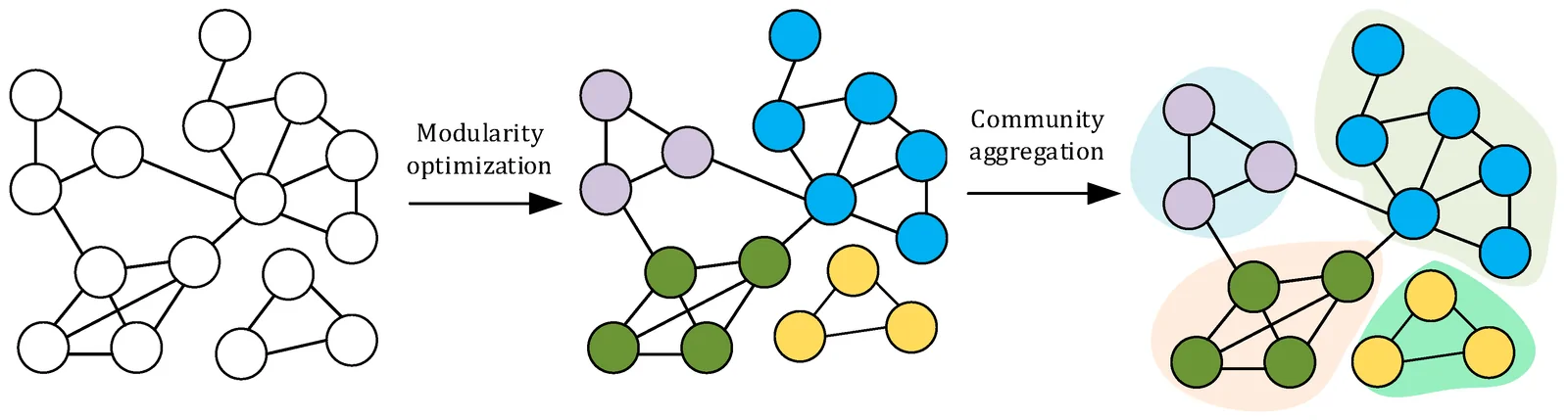
Schematic diagram of community detection of alliance network.

More critically, community detection divides the entire network into multiple sub-networks, creating network boundaries for different communities, where nodes within each sub-network are relatively more interconnected, and interconnections between different sub-networks are sparser. This extraction of sub-networks enhances the granularity of research, offering deeper insights. The Louvain method of community detection is an efficient approach suited for handling large-scale network data, aiding researchers in identifying heterogeneous community groups within networks [55]. Its process includes two processes, modularity optimization and community aggregation, as shown in Figure 2. Different colors represent different network communities. This methodological approach underscores the notion that not all nodes contribute equally to the network’s functionality, and it is the formation of these tight-knit communities that facilitate the exchange of ideas and resources critical for innovation [56]. Its primary objective is to maximize modularity, an indicator that measures the quality of community division as follows:

(8) $$Modularity=\frac{1}{2m}\sum_{i,j}\left[A_{ij}-\frac{k_{i}k_{j}}{2m}\right]\delta \left(c_{i},c_{j}\right)$$

where $A_{ij}$ denotes the element of the adjacency matrix. $k_{i}$ and $k_{j}$ are the degrees of nodes of *i* and *j*, respectively. *m* is the total number of edges. $\delta \left(c_{i},c_{j}\right)$ is the Kronecker delta function, which equals 1 if nodes *i* and *j* are in the same community and 0 otherwise.

#### 3.3.2. Machine Learning

In the context of delineated network communities, each enterprise node constitutes a member of its respective community, with each community exhibiting distinct network structures [57]. This distinction manifests in network size, density, and average path length, representing typical high-dimensional data. This study adopts an exploratory framework rather than predefining explicit hypotheses, thus prioritizing the identification of hidden patterns and rules within the data and determining the network embedding conditions that drive high innovative performance. K-Means, as an unsupervised learning method, effectively reveals the potential network structures within communities and the similarities across different communities. Consistent with the existing division principle, “birds of a feather flock together”, it allows for the grouping of communities with similar structures into the same environmental category [58]. Compared to other clustering methods such as hierarchical clustering, AP clustering, and DBSCAN, K-Means offers greater interpretability and performs better in clustering convex data like network indicators.

The classification and regression tree (CART), as a supervised machine learning method, segments data into various categories or outcomes through a series of decision nodes [23], further exploring the potential associative rules between different network embedding conditions and innovation performance. To construct the binary innovation-performance outcome required for the classification analysis, *PE* is initially divided into high and low performance categories using its sample mean as the baseline cutoff. The mean is adopted because it provides a transparent and reproducible criterion for distinguishing relatively higher and lower innovation performance without imposing an arbitrary percentile-based threshold.

The basic modeling steps are as follows:

Step 1: Determine if the innovation performance category under a certain feature is pure. If so, directly generate the dependency relation between that feature and performance level, forming a single-node decision tree. If not, proceed to the next step.

Step 2: Calculate the *Gini* coefficient, an entropy-based degree of data uncertainty for the current sample *D*.

(9) $$Gini\left(D\right)=1-{\sum_{k=1}^{K}\left(\frac{\left|C_{k}\right|}{\left|D\right|}\right)}^{2}$$

where *K* is the number of categories and *C_k_* is the number of samples under category *K*.

Step 3: Calculate the *Gini* coefficient for each feature within the current sample, selecting the feature and feature value that minimize the *Gini* result to divide the dataset into two parts, *D*_1_ and *D*_2_, and establish the left and right nodes of the decision tree. The *Gini* coefficient under feature *A* is calculated as

(10) $$Gini\left(D,A\right)=\frac{\left|D_{1}\right|}{\left|D\right|}Gini\left(D_{1}\right)+\frac{\left|D_{2}\right|}{\left|D\right|}Gini\left(D_{2}\right)$$

Step 4: Recursively invoke Steps 1 to 3 on the left and right child nodes to generate the decision tree.

Step 5: Set pruning rules for the generated decision tree. If the proportion of samples in a node relative to the total samples is less than the threshold *Support* or if the *Gini* coefficient of the samples exceeds the threshold *Confidence*, halt the generation of further decision trees.

CART was selected as the primary model for configurational analysis because its recursive partitioning structure produces explicit and transparent decision paths, allowing the threshold conditions and interactions among network embedding features to be directly identified and interpreted. In contrast, ensemble methods such as Random Forest and XGBoost aggregate a large number of optimized trees and can achieve strong predictive performance, but their resulting decision logic is substantially less transparent when the research objective is to identify specific conditional configurations. In addition, CART provides a relatively transparent bridge between predictive modeling and subsequent rule extraction. The resulting decision paths can be further summarized using *Support* and *Confidence* to identify representative conditional combinations associated with different innovation performance states.

## 4. Results

### 4.1. Descriptive Statistics

The descriptive statistics and correlation analysis provided in Table 2 offer a detailed overview of the variables of this study. To prevent the interference of outliers in the original sample with the results of data analysis, we applied a trimming technique, removing extreme values in the upper and lower 1% tails while retaining the sample size. This step effectively cleansed the data of any outliers. Additionally, we computed the Pearson correlation coefficients between each variable and the basic descriptive statistics for each variable. The results revealed that there is a significant positive correlation between *CB* and *SH*, suggesting that firms with a broader range of alliances tend to occupy positions that bridge structural holes in the network. Firm innovation performance showed weak negative correlations with both cooperation breadth and structural holes, suggesting that there may not be a simple linear relationship between network characteristics and firm innovation.

Furthermore, the correlation coefficients between all other variables were also below 0.6. Moreover, we calculated the variance inflation factors and found that all the results were below 2, far less than 10. Therefore, we can conclude that there is no presence of multicollinearity. These descriptive statistics and correlation results lay the groundwork for a deeper investigation into how various dimensions of network embeddedness influence corporate innovation. The observed correlations provide preliminary insights into the complex interplay between structural and relational embeddedness and innovation outcomes, underscoring the importance of considering multiple facets of network dynamics in understanding innovation performance.

### 4.2. Division of Alliance Network Typology

Firms are embedded in alliance networks with substantially different structural characteristics, which may lead to heterogeneous innovation outcomes. To identify these differences, this study first applies the Louvain community detection algorithm to each alliance network and identifies densely connected sub-communities. For each community, five topological characteristics—network size, average clustering coefficient, network density, network diameter, and average path length—are then calculated to characterize its structural configuration.

**Figure 3:**
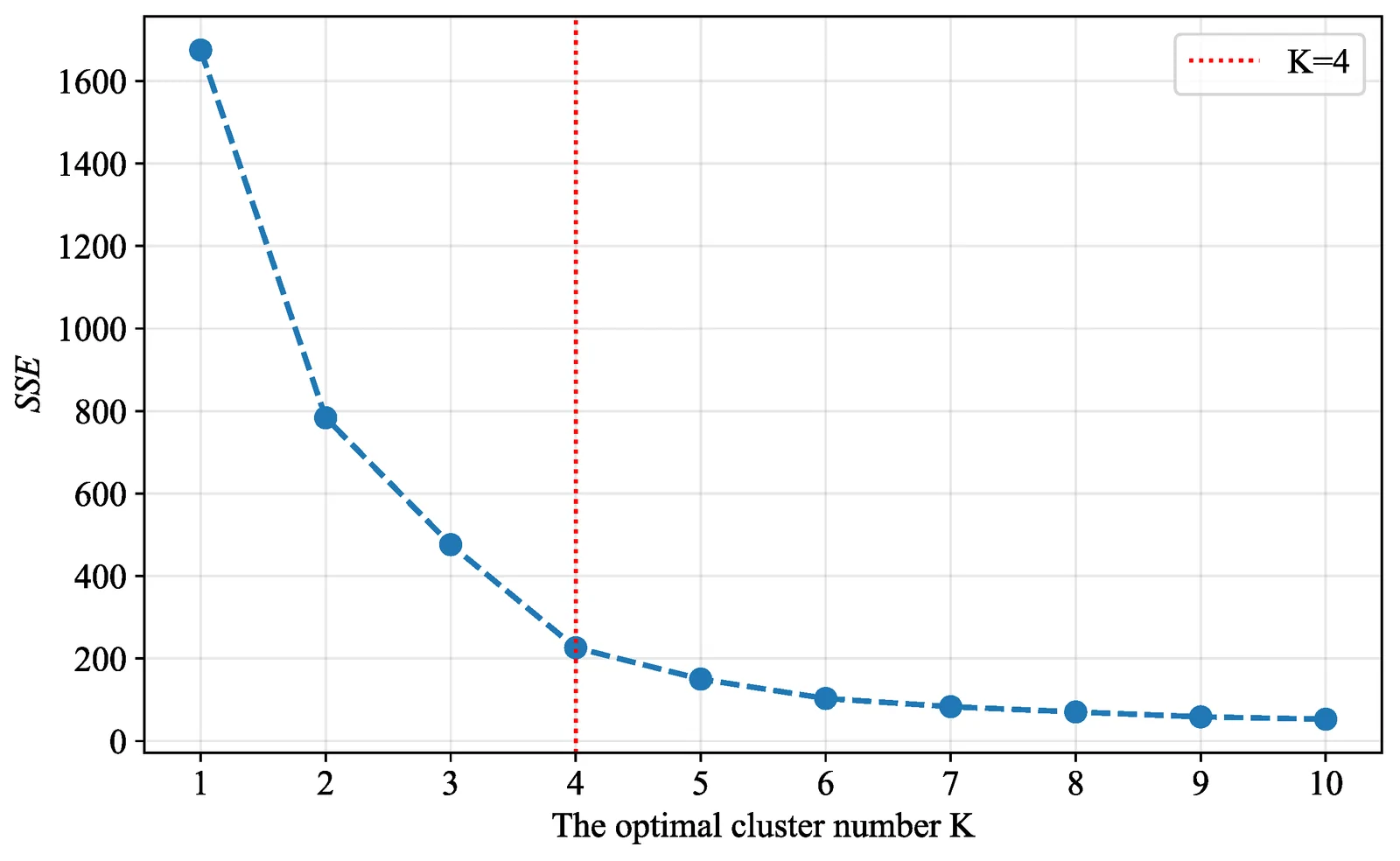
Determination of the optimal number of clusters using the elbow method.

Next, the K-Means clustering algorithm is employed to classify the community networks according to these topological characteristics. The optimal number of clusters is determined using the elbow method as shown in Figure 3. The sum of squared errors (SSE) decreases substantially as the number of clusters increases up to four, whereas the reduction in SSE becomes considerably smaller when the number of clusters exceeds four. This indicates that adding further clusters provides only limited improvement in within-cluster homogeneity. Therefore, K = 4 is selected as the clustering solution, providing a reasonable balance between within-cluster similarity and between-cluster heterogeneity.

**Figure 4:**
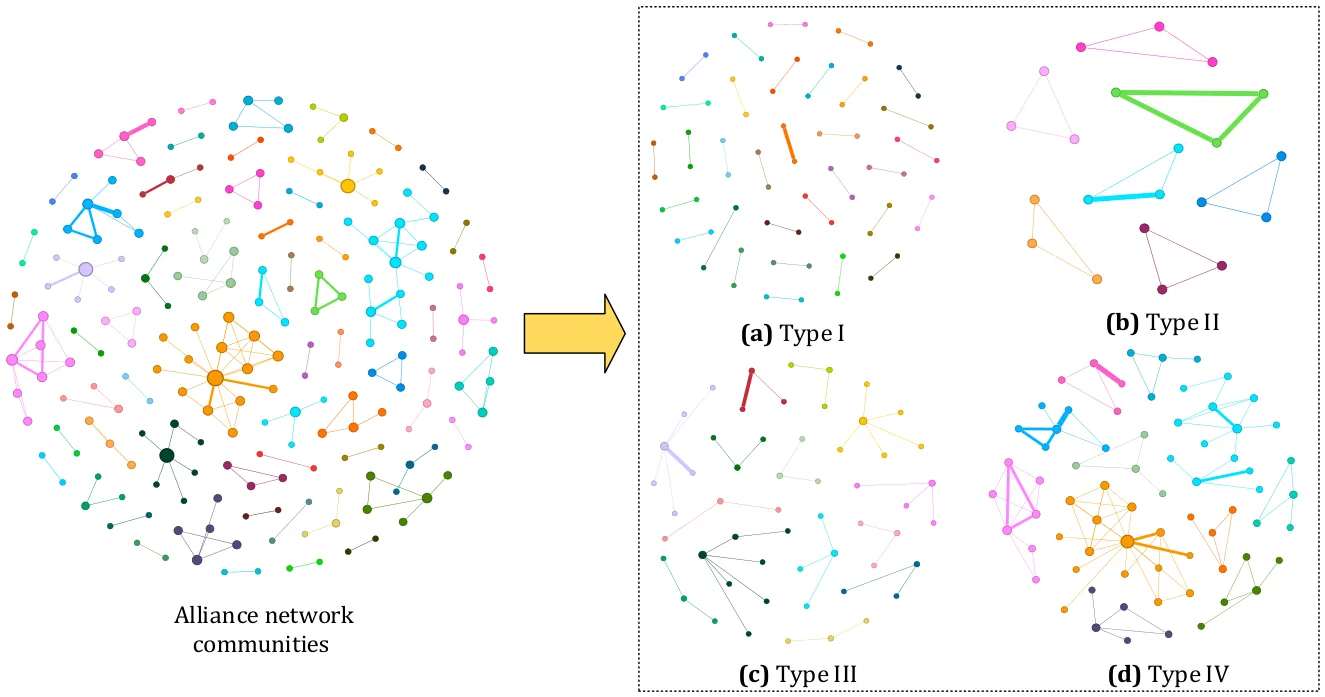
The division of specific alliance network communities.

Based on their distinctive structural characteristics, the four clusters are labeled as dyadic, ringlike, star, and complex alliances, respectively. This classification captures differences in the structural environments in which firms are embedded and provides the basis for subsequent configurational analysis. Figure 4 illustrates the community division and network typology identification using the 2018–2020 alliance network as an example. To examine the temporal stability of the classification, the clustering procedure is further applied across the three-year rolling windows from 2008–2010 to 2018–2020. Although the number of focal firms assigned to each type varies across periods, the four major structural patterns remain consistently identifiable, with their characteristic topological features broadly preserved. This indicates that the typology is not driven by a particular observation period and provides a relatively stable basis for subsequent analysis.

Table 3 summarizes the overall network characteristics of the four alliance types. Type I is characterized by relatively simple dyadic connections, Type II by a ringlike structure, Type III by a star-shaped configuration, and Type IV by a more complex combination of network structures. These distinct structural configurations provide the basis for examining whether the association between network embedding and innovation performance varies across alliance network types.

Dyadic alliance networks represent the simplest form of cooperation, where a firm engages in a one-to-one partnership. This network type, grounded in resource dependence theory, emphasizes direct, manageable relationships but may limit access to diverse resources and information flows [59]. Ringlike alliance networks manifest through firms interconnected in a closed loop, facilitating equal information exchange and collaboration. The structure, supported by the strength of weak ties theory [11], promotes balanced cooperation but requires concerted coordination efforts. Furthermore, star alliance networks feature a central firm connected to multiple partners, embodying the structural holes theory and centrality concepts. The central firm controls resource distribution and information flow, optimizing network utility and fostering innovation through bridging diverse groups. Finally, complex alliance networks combine elements of ringlike and star configurations, showcasing high diversity and complexity. According to complexity theory, such networks leverage the advantages of various structures, offering rich social capital and broad knowledge acquisition channels, albeit at the cost of increased management challenges [60]. The heterogeneous division not only aligns with previous theoretical perspectives but also provides a practical framework for understanding how different network configurations influence resource access, collaboration efficiency, and innovative capacity.

### 4.3. Decision Rules Analysis

To evaluate the predictive performance of alternative machine learning algorithms across heterogeneous alliance network types, this study compares four representative classification models: CART, Random Forest, XGBoost, and LightGBM. The comparison serves as an empirical benchmarking step before extracting interpretable decision rules. CART constructs decision trees through recursive binary partitioning, whereas Random Forest, XGBoost, and LightGBM employ ensemble learning strategies to improve predictive performance through the aggregation or sequential optimization of multiple tree-based learners.

For CART, the maximum tree depth is set to 5 layers to balance model complexity and interpretability. Random Forest is specified with 100 trees (n_estimators = 100), while XGBoost and LightGBM are likewise configured with 100 base learners. All models are initialized with a fixed random seed (random_state = 42) to ensure experimental reproducibility. The key hyperparameters of the four classification models are reported in Table 4.

Regarding the validation strategy, the study adopts stratified 5-fold cross-validation to assess the generalization performance of the models. Specifically, the sample data are partitioned into five subsets according to the class-proportion strata, and each fold iteratively uses four subsets as the training set and the remaining one as the test set for model training and evaluation. To avoid information leakage during cross-validation, a new model instance is fitted independently within each training fold.

**Figure 5:**
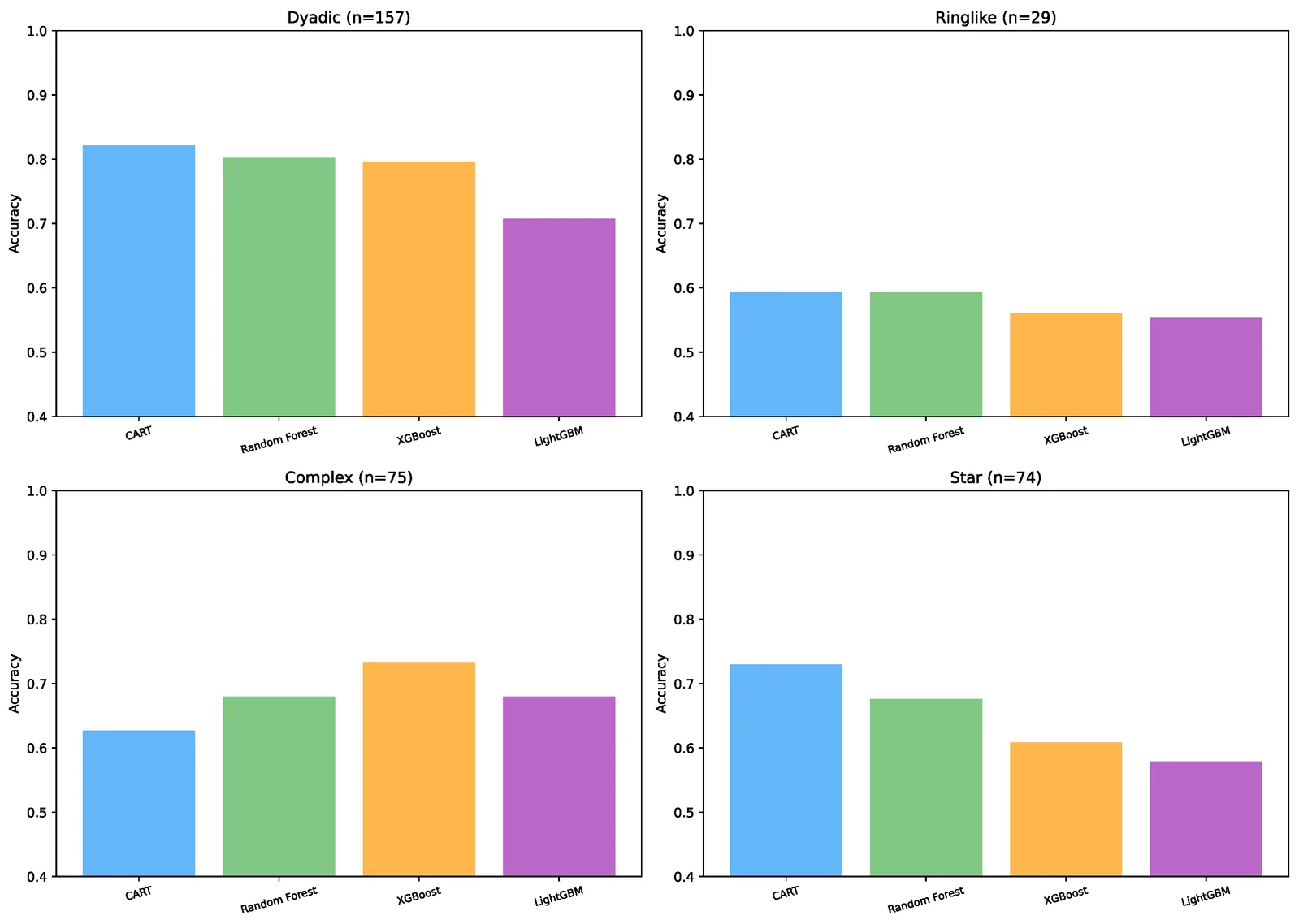
Model Comparison by Alliance Type.

Model performance is evaluated using accuracy and F1-score. Accuracy captures the overall proportion of correctly classified observations, whereas F1-score provides a complementary assessment by jointly considering precision and recall. The comparative results for each alliance network type are reported in Table 5 and Figure 5.

As shown in Table 5, the relative predictive performance of the four machine learning models varies across alliance network types. CART achieves the highest accuracy and F1-score for the dyadic, ringlike, and star alliance types, whereas XGBoost performs better for the complex alliance type. These results indicate that the predictive advantage of a particular algorithm is contingent on the underlying alliance network structure, and no single model uniformly dominates across all network types.

Importantly, the model comparison does not imply that CART has superior predictive performance in every subgroup. Rather, the results provide an empirical benchmark for subsequent model selection. Although XGBoost achieves better predictive performance for the complex alliance type, CART is retained as the primary model for the subsequent decision-rule analysis because the central objective of this study is to identify interpretable conditional combinations of network embedding features associated with innovation performance. CART provides explicit hierarchical decision paths and threshold conditions that can be directly translated into interpretable rules [22,23], whereas the ensemble structure of XGBoost, despite its predictive advantages, makes the extraction of individual configurational pathways less transparent.

**Figure 6:**
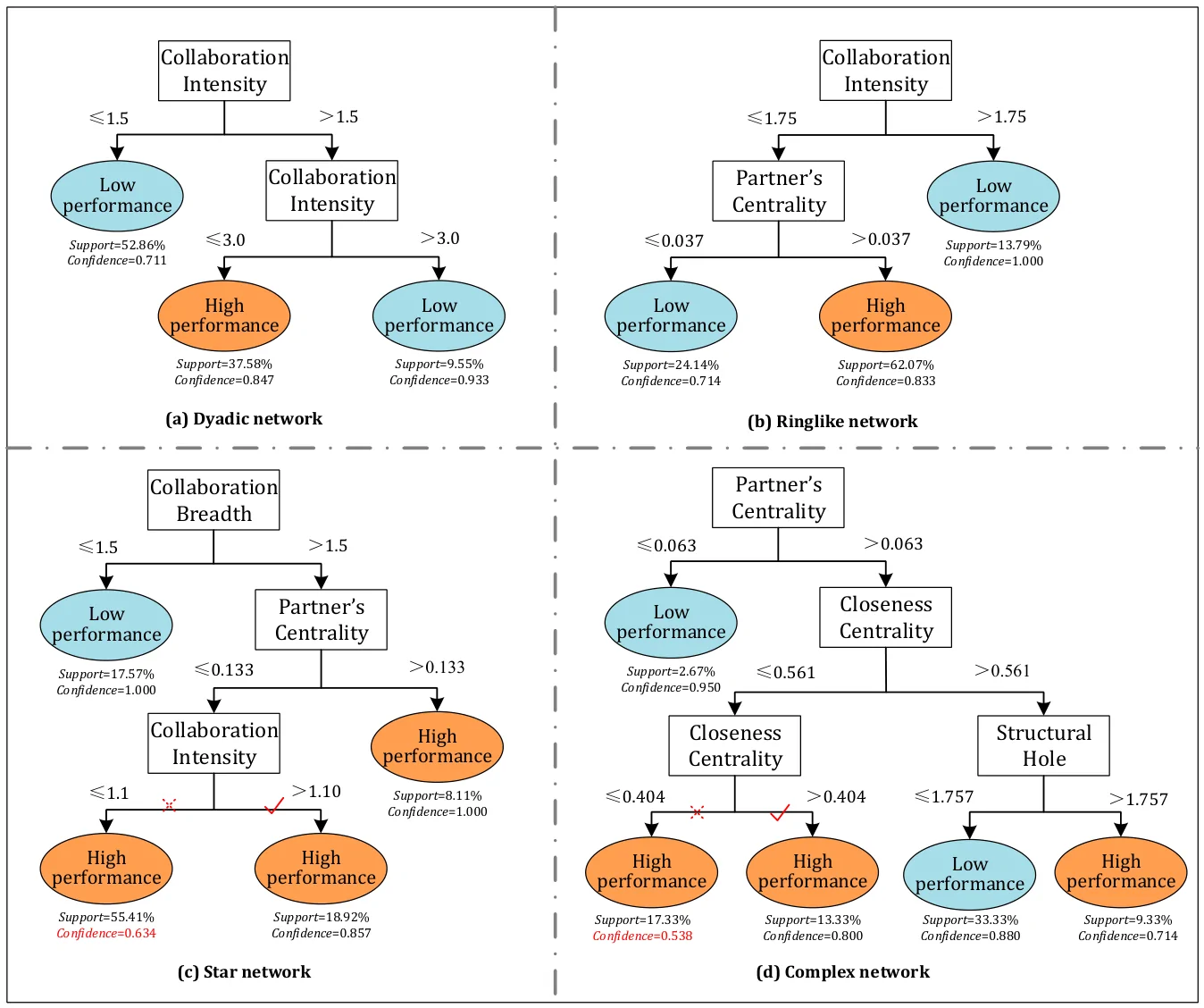
Visualization of decision trees for diverse network types.

Accordingly, the selection of CART reflects a balance between predictive performance and configurational interpretability rather than a claim that CART universally outperforms other machine learning algorithms. The alternative models are retained as predictive benchmarks, while CART is used to generate the interpretable decision rules reported in Figure 6 and Table 6. Rather than imposing a fixed Support threshold across alliance types, this study retains representative decision rules identified by the CART models. Support is reported to indicate the proportion of observations covered by each rule, while rules with a Confidence value of 0.70 or above are retained for interpretation.

The decision rules reveal clear differences in the configurational associations between alliance network embedding and innovation performance across the four alliance types. In dyadic alliances, cooperation intensity is the only condition identified. A moderate *CI* (1.5 < *CI* < 3.0) is associated with high innovation performance, whereas both lower and higher *CI* correspond to low performance. This non-monotonic pattern suggests that the innovation value of relational embeddedness is contingent on the intensity of interaction: limited interaction may constrain knowledge exchange, whereas excessive interaction may coincide with greater relational redundancy or coordination demands. Thus, dyadic embeddedness does not exhibit a uniformly positive association with innovation.

In ringlike alliances, the association becomes conditional on both *CI* and partner centrality. When *CI* ≤ 1.75, higher partner centrality is associated with high performance, whereas lower partner centrality corresponds to low performance; *CI* > 1.75 is associated with low performance. This indicates that the value of relational intensity is intertwined with who occupies the relevant network position, consistent with the network-embeddedness view that access to valuable knowledge depends not only on relationship strength but also on partners’ structural positions.

The star and complex alliances show more multidimensional configurations. In star alliances, cooperation breadth is associated with performance together with partner centrality and, under certain conditions *CI*. In complex alliances, partner centrality, closeness-related position, and structural holes jointly appear in the decision rules. Compared with dyadic alliances, these results indicate that as network structures become more complex, no single embedding characteristic is sufficient to characterize innovation-performance differences; rather, the association depends increasingly on combinations of relational and structural conditions.

Overall, the results provide a configurational explanation for the inconsistent innovation outcomes associated with alliance network embedding. The four network types exhibit different decision thresholds and combinations, indicating that the innovation value of embeddedness is contingent on network structure and the configuration of multiple embedding characteristics, rather than determined by a universally beneficial level of any single network attribute. In this respect, CART complements the configurational perspective by identifying specific thresholds and conditional decision rules within different network types, while these rules should be interpreted as predictive associations rather than causal effects.

### 4.4. Robustness Test

To examine whether the configurational associations identified by the decision-tree analysis are sensitive to the choice of analytical method, this study further applies fuzzy-set qualitative comparative analysis [16] to the four alliance network types. Unlike CART, which identifies threshold-based decision rules, fsQCA examines whether combinations of conditions are sufficient for high or non-high innovation performance. The results are reported in Table 7.

Overall, the fsQCA results provide support for several core configurational patterns identified by CART, while also revealing some alternative configurations. The strongest correspondence appears in dyadic alliances, where *CI* remains the key condition in the high-performance configuration H1b. In ringlike and star alliances, fsQCA retains several conditions identified by CART but combines them differently. The most notable discrepancy occurs in complex alliances. While CART associates high innovation performance with the combination of high *CC*, high *PC*, and high *SH* (H4b), fsQCA identifies an alternative high-performance configuration characterized by the presence of *CB* and the absence of *CC*, *PC*, and SH (H4b; consistency = 0.833; raw coverage = 0.349). fsQCA also identifies alternative non-high configurations that are not directly reproduced by the corresponding CART rules. These differences reflect the distinct analytical logics of the two methods: CART identifies hierarchical, threshold-based decision rules, whereas fsQCA identifies sufficient combinations of conditions. Thus, the cross-method results support the robustness of the configuration-dependent and network-type-specific association between alliance network embedding and innovation performance, while also revealing alternative configurational pathways that may not be captured by a single decision-tree structure.

Moreover, considering that the baseline analysis dichotomizes *PE* using the sample mean, we conduct an additional robustness check by replacing the mean cutoff with the median. The resulting decision trees show some minor changes in the specific splitting thresholds of individual variables, which is expected because the alternative cutoff changes the classification of innovation-performance outcomes. Importantly, however, the key network embedding variables appearing in the decision rules and the main conditional combinations associated with high innovation performance remain largely unchanged. Thus, the substantive configurational patterns are not materially affected by whether the mean or median is used as the cutoff.

## 5. Discussion

The findings of this study provide a configurational perspective for understanding why alliance network embeddedness is associated with heterogeneous innovation outcomes. Rather than treating network characteristics as isolated determinants, the results indicate that their relevance depends on the structural context and their combination with other embedding conditions. This perspective is broadly consistent with prior research on network embeddedness and innovation, while providing a more differentiated interpretation of the apparent inconsistency in existing findings [21,23].

A first important finding is the existence of four distinct alliance network types, dyadic, ringlike, star, and complex alliances. This result complements conventional network analyses that primarily focus on individual firms’ positions or overall network characteristics [61]. From the perspective of network embeddedness, firms do not operate in structurally identical relational environments. The structure of connections surrounding a focal firm determines how resources, knowledge, and information can be accessed and recombined.

The four network types identified in this study are broadly consistent with established perspectives on interorganizational relationships. Dyadic structures represent relatively direct and concentrated cooperation, whereas ringlike structures emphasize interconnected and closed relationships. Star structures are characterized by a more prominent central position and bridging relationships, while complex structures combine multiple relational and structural features. These distinctions are compatible with the theoretical insights of Granovetter [11], Burt [47], Podolny and Page [13] which emphasize that the value of network relationships depends on the structure of connections rather than simply on the existence of ties.

This finding also helps explain why previous studies sometimes report different effects for similar network characteristics [21]. Research focusing on a single network indicator may implicitly assume that firms face comparable structural environments [15]. However, the present results suggest that the same level of cooperation intensity, partner centrality, or structural-hole position can have different implications depending on the broader network configuration. Thus, network type can be understood as an important contextual condition for interpreting the value of network embeddedness rather than merely as a descriptive classification.

The decision-tree results further show that the association between network embedding and innovation performance is not adequately represented by a single dominant network characteristic. More importantly, the relevant conditions differ across the four alliance network types. In dyadic alliances, cooperation intensity is the dominant condition, and a moderate range is associated with high innovation performance. This is broadly consistent with prior research emphasizing the role of interfirm interaction in facilitating knowledge and resource exchange [6,62]. At the same time, the threshold pattern suggests that the innovation relevance of cooperation intensity is not simply monotonic. In a relatively simple network structure, the extent of interaction itself becomes particularly salient.

In ringlike alliances, however, cooperation intensity is associated with innovation performance together with partner centrality. This finding is consistent with the argument that firms benefit not only from the intensity of their relationships but also from the resources and positions of their partners [63]. It also resonates with the notion of second-order social capital discussed by Jiang et al. [49]. The implication is that the value of a relationship may depend on the structural position through which external knowledge and resources are accessed.

The star and complex alliance results further reinforce this contextual logic. In star alliances, cooperation breadth and partner position become more relevant, whereas complex alliances involve several structural and relational conditions. This is broadly consistent with research emphasizing the role of structural holes and advantageous network positions in innovation [7]. However, the present findings suggest that these characteristics should not be interpreted independently: their association with innovation performance depends on the surrounding configuration.

The findings also contribute to the ongoing discussion concerning whether network characteristics operate independently, complementarily, or as substitutes. Previous research has examined mechanisms such as network density, structural holes, cooperation breadth, and centrality separately or has emphasized particular forms of complementarity and substitution [19]. The present results suggest that these relationships are more conditional than a simple classification into “complementarity” or “substitution” would imply.

For example, in ringlike alliances, partner centrality becomes particularly relevant when cooperation intensity is relatively low. In complex alliances, the innovation-performance association depends on combinations involving structural and relational conditions. These patterns indicate that the contribution of one network characteristic can change according to the state of other characteristics. Importantly, this does not establish a causal substitution or complementarity mechanism. Rather, it reveals configurational associations that are consistent with the possibility that different network conditions can compensate for, reinforce, or constrain one another.

This finding is particularly relevant to the literature on configurational approaches to organizational research. Reck and Fliaster [16], for example, emphasize that organizational outcomes can emerge from combinations of conditions rather than from isolated variables. The present study extends this perspective to alliance network embeddedness by showing that the relevant configurations are themselves network-type-specific. A configuration associated with high innovation performance in a dyadic alliance does not necessarily apply to a star or complex alliance. Consequently, the results support a move from a general search for an “optimal” network position toward a more contextual understanding of how firms combine relational and structural resources.

## 6. Conclusions

### 6.1. Main Conclusions

This study examines how alliance network embeddedness is associated with corporate innovation performance from a configurational perspective. Based on 335 observations of Chinese biopharmaceutical manufacturing firms, the study combines community detection, K-means clustering, and machine learning-based decision-tree analysis to identify heterogeneous alliance network structures and their associated innovation-performance configurations. The main conclusions are as follows.

First, alliance networks exhibit distinct structural configurations rather than a homogeneous embedding pattern. The analysis identifies four major types of alliance networks: dyadic, ringlike, star, and complex alliances. These types differ in their structural connectivity, centrality, density, and relational characteristics, indicating that firms are embedded in qualitatively different network environments. The cross-period clustering analysis further supports the stability of this four-type typology across the rolling observation windows.

Second, the association between alliance network embedding and innovation performance is configuration-dependent. The decision-tree analysis shows that no single network embedding characteristic consistently corresponds to high innovation performance across all alliance types. Instead, different network structures exhibit distinct decision rules. In dyadic alliances, cooperation intensity presents a clear threshold pattern, with moderate intensity associated with high innovation performance. In ringlike alliances, cooperation intensity and partner centrality jointly distinguish innovation-performance outcomes. In star alliances, central network position and alternative combinations of network characteristics are particularly important. Complex alliances exhibit more heterogeneous combinations involving multiple structural and relational conditions. These findings indicate that the value of network embedding depends on the specific structural context in which firms are positioned.

Third, the results reveal threshold effects and alternative configurational pathways. The decision rules demonstrate that the relationship between network characteristics and innovation performance cannot be adequately represented by a simple linear relationship. For example, both insufficient and excessive cooperation intensity may be associated with lower innovation performance in dyadic alliances, whereas different combinations of network characteristics can correspond to similar performance outcomes in more complex structures. Thus, firms may reach similar innovation outcomes through different configurations rather than through a universally optimal network position. The robustness analyses support the configurational interpretation while also revealing complementary pathways.

Overall, the study concludes that the innovation value of alliance network embedding is contingent on network type and the configuration of multiple network characteristics. Rather than seeking a universally superior network position, firms need to align their network structure and relational strategies with the specific characteristics of the alliance environment in which they are embedded.

### 6.2. Implications

#### 6.2.1. Theoretical Implications

This study offers several theoretical implications. First, it extends the understanding of alliance network embeddedness by highlighting its configurational and context-dependent nature. Rather than treating individual network characteristics as having stable effects on innovation, the findings show that their associations with innovation performance depend on the specific alliance network structure in which firms are embedded. This provides a more differentiated explanation for the heterogeneous innovation outcomes associated with alliance network embeddedness.

Second, the study highlights the importance of network typology in understanding the value of embeddedness. The different decision rules identified for dyadic, ringlike, star, and complex alliances suggest that the same network characteristic may have different implications under different structural conditions. Thus, network structure provides an important contextual condition for interpreting the relationship between embeddedness and innovation.

Third, the study demonstrates the complementary value of machine-learning-based decision rules for configurational research. CART identifies interpretable thresholds and hierarchical conditional pathways from continuous network characteristics, while fsQCA captures alternative sufficient combinations. Their complementary use provides a more detailed representation of configurational associations than relying exclusively on individual net effects.

#### 6.2.2. Practical Implications

The findings also provide several practical implications for firms managing alliance networks. First, firms should avoid pursuing a universally optimal level of network embeddedness. Appropriate network strategies should be matched with the structural characteristics of the alliance network in which the firm is positioned.

Second, firms can use the identified conditional thresholds as reference points when adjusting alliance strategies. For example, in dyadic alliances, moderate cooperation intensity is associated with higher innovation performance, whereas in ringlike, star, and complex alliances, innovation performance depends more strongly on combinations of network position, partner characteristics, and relational conditions.

Third, alliance management should focus on configuring multiple network conditions rather than optimizing a single indicator. Firms can adjust partner selection, cooperation intensity, network breadth, and structural positioning according to their specific network type, thereby improving the alignment between alliance structure and innovation objectives. Importantly, these decision rules should be regarded as empirically observed configurational associations rather than causal prescriptions, and should therefore serve as reference points rather than deterministic managerial rules.

### 6.3. Limitations and Future Research

This study has several limitations. First, the sample is restricted to Chinese biopharmaceutical manufacturing firms, a highly R&D-intensive and knowledge-intensive industry. The identified configurational patterns may therefore not be directly generalizable to other industries with different innovation and alliance characteristics. In addition, the study relies on CSMAR, CNRDS, and Innojoy databases, whose coverage and classification of corporate, alliance, and patent information may impose certain constraints on the measurement of network embedding and innovation performance. Future research could extend the analysis to other industries and countries and incorporate alternative data sources to examine the boundary conditions and generalizability of these findings.

Second, the decision-tree rules identify configurational associations rather than causal effects. Although lagged innovation performance and robustness tests are employed, the study does not establish causal relationships between network embedding and innovation performance. Future research could incorporate structural equation models [64], case study [65] or longitudinal causal designs to further examine these relationships.

## Figures and Tables

**Table 1:** Variables mentioned and their descriptions.

Variable Type	Symbol	Description
Dependent variable	*PE*	Firm innovation performance. It is comprehensively evaluated through both the quality and quantity of patents. The quality metrics of a patent include number of claims, forward citations, non-patent literature citations, IPC numbers and grant status. The weights for each of these quality indicators are determined using the entropy weighting method.
Network embedding features	*SH*	Structure hole. It refers to the gaps in a social network where there is an absence of direct ties between two parts of the network (structure embeddedness).
	*CC*	Closeness centrality. It measures how close a firm is to all other firms within the network, calculated as the inverse of the average length of the shortest paths from the firm to all other firms in the network (structure embeddedness).
	*PC*	Partner’s centrality. It assesses the centrality of a firm’s partners within the network. It reflects the notion that not all partners are equal; having central partners can provide strategic advantages (structure embeddedness).
	*CB*	Cooperation breadth. It refers to the diversity and range of a firm’s collaborative engagements across different partners within the network (relational embeddedness).
	*CI*	Cooperation intensity. It measures the depth and strength of a firm’s collaborative relationships with its partners (relational embeddedness).
Cluster variables	*NS*	Network size. This metric represents the total number of nodes within a community network. It quantifies the community’s scale.
	*ACC*	Average clustering coefficient. This coefficient measures the degree of clustering within the community network, indicating how closely knit groups of nodes are. A higher coefficient suggests that nodes tend to form tightly interconnected groups.
	*NDE*	Network density. This metric evaluates the level of connectivity within the community network by comparing the actual links among nodes to the maximum possible links. Higher network density indicates a more interconnected community, potentially facilitating easier and more efficient resource and information flow.
	*NDI*	Network diameter. It refers to the longest of all the shortest paths between any two nodes within the community network. It offers insights into the “spread” or maximum separation between nodes in the community network.
	*APL*	Average path length. This metric calculates the average number of steps along the shortest paths for all possible pairs of nodes within the community network.

**Table 2:** Descriptive statistics and correlation analysis results.

	Mean	Std	Median	Min	Max	*PE*	*CB*	*CI*	*BC*	*CC*	*PC*
*PE*	2.017	0.882	1.810	0.438	7.748	1.000					
*CB*	2.045	1.612	1.000	1.000	14.000	−0.126	1.000				
*CI*	1.172	0.617	1.000	1.000	9.000	0.00	−0.122	1.000			
*SH*	1.211	0.305	1.000	0.875	1.834	−0.045	0.578 *	−0.169 *	1.000		
*CC*	0.910	0.206	1.000	0.202	1.000	0.122	−0.058	0.1300	−0.189 *	1.000	
*PC*	0.095	0.202	0.023	0.004	1.000	0.009	0.5814 *	−0.039	0.323 *	−0.096	1.000

*Note:* * indicates that the two corresponding variables are significantly correlated.

**Table 3:** The overall network metrics for heterogeneous alliance network types.

Type	Firm Number	Average Clustering Coefficient	Average Path Length	Average Diameter	Average Network Density	Label Names
Type I	157	0.000	1.000	1.000	1.000	Dyadic alliance
Type II	29	1.000	1.000	1.000	1.000	Ringlike alliance
Type III	74	0.000	1.520	2.243	0.531	Star alliance
Type IV	75	0.342	1.989	3.453	0.390	Complex alliance

**Table 4:** Hyperparameter Settings of the Four Machine Learning Models.

Hyperparameter	CART	Random Forest	XGBoost	LightGBM
Number of estimators	—	100	100	100
Criterion	*Gini*	*Gini*	Log-loss	Binary log-loss
Maximum depth	5	None	5	5
Minimum sample split	5	5	—	—
Minimum sample leaf	1	1	—	—
Learning rate	—	—	0.1	0.1
Random state	42	42	42	42

Note: “—” indicates that the hyperparameter is not applicable to the corresponding model.

**Table 5:** Best Model selection per Subgroup.

Alliance Type	N	Best by Accuracy	Accuracy	Best by F1	F1
Dyadic	157	CART	0.8218	CART	0.8171
Ringlike	29	CART	0.5933	CART	0.5719
Complex	75	XGBoost	0.7333	XGBoost	0.7258
Star	74	CART	0.7295	CART	0.6952

**Table 6:** Crucial decision rules for various alliance network types.

Network Type	Conditional Factor					Criteria		Decision Factor	Key Rule
	*CB*	*CI*	*CC*	*PC*	*SH*	*Support*	*Confidence*	*PE*	Index
Dyadic alliance *N* = 157 (Low: High = 1.12)	-	≤1.5	-	-	-	52.86%	0.711	Low	H1a
	-	(1.5, 3.0)	-	-	-	37.58%	0.847	High	H1b
	-	>3.0	-	-	-	9.55%	0.933	Low	H1c
Ringlike alliance *N* = 29 (Low: High = 0.71)	-	≤1.75	-	>0.037	-	62.07%	0.833	High	H2a
	-	≤1.75	-	≤0.037	-	24.14%	0.714	Low	H2b
	-	>1.75	-		-	13.79%	1.000	Low	H2c
Star alliance *N* = 74 (Low: High = 0.72)	>1.5	-	-	>0.133	-	8.11%	1.000	High	H3a
	≤1.5	-	-		-	17.57%	1.000	Low	H3b
	>1.5	>1.1	-	≤0.133	-	18.92	0.857	High	H3c
Complex alliance *N* = 75 (Low: High = 2.26)	-	-	-	≤0.063	-	2.67%	0.950	Low	H4a
	-	-	>0.561	>0.063	>1.757	9.33%	0.714	High	H4b
	-	-	>0.561	>0.063	≤1.757	33.33%	0.880	Low	H4c
	-	-	(0.404, 0.561)	≤0.063	-	13.33%	0.800	High	H4d

**Table 7:** The fsQCA results for diverse alliance types.

	Dyadic Alliance		Ringlike Alliance		Star Alliance		Complex Alliance		
Conditions	High	Non-High	High	Non-High	High	Non-High	High	Non-High1	Non-High2
*CB*				●	◊	×	●	●	●
*CI*	●	◎	◎	◊					×
*CC*			●	●	●	◎	×	◊	
*PC*			◊		◊	●	◊	●	●
*SH*			×		●	●	◊	●	
Consistency	0.845	0.669	0.818	0.933	0.847	0.781	0.833	0.849	0.853
Raw coverage	0.594	0.801	0.174	0.274	0.619	0.176	0.349	0.502	0.631
Unique coverage	0.594	0.801	0.001	0.074	0.069	0.175	0.349	0.080	0.209
Rules	H1b	H1a	H2a	H2c	H3a	H3b	H4b	H4a	H4c

Notes: ◊ indicates the presence of a core condition, ◎ indicates the absence of a core condition, ● indicates the presence of a borderline condition, × indicates the absence of a borderline condition, and “blank” indicates that the presence or absence of the condition is irrelevant to the outcome.

## Data Availability

Data in this study are available from the corresponding author upon reasonable request.
